# Postharvest NMR Metabolomic Profiling of Pomegranates Stored Under Low-Pressure Conditions: A Pilot Study

**DOI:** 10.3390/metabo15080507

**Published:** 2025-07-30

**Authors:** Keeton H. Montgomery, Aya Elhabashy, Brendon M. Anthony, Yong-Ki Kim, Viswanathan V. Krishnan

**Affiliations:** 1 Department of Chemistry and Biochemistry, California State University, Fresno, CA 93740, USAaya9998@mail.fresnostate.edu (A.E.); 2 Department of Fruit Sciences, RipeLocker, Inc., Bainbridge Island, WA 98110, USA; brendon@ripelocker.com (B.M.A.);; 3 Department of Pathology and Molecular Medicine, University of California Davis School of Medicine, Sacramento, CA 95817, USA

**Keywords:** low pressure, hypobaric storage, metabolome, mass spectrometry, fruit quality, *Punica granatum* L.

## Abstract

Background: There is a high demand for long-term postharvest storage of valuable perishables with high-quality preservation and minimal product loss due to decay and physiological disorders. Postharvest low-pressure storage (LPS) provides a viable option for many fruits. While recent studies have presented the details of technology, this pilot study presents the metabolomics changes due to the hypobaric storage of pomegranates as a model system. Methods: Nuclear magnetic resonance (NMR)-based metabolomics studies were performed on pomegranate fruit tissues, comparing fruit stored under LPS conditions versus the traditional storage system, with modified atmosphere packaging (MAP) as the control. The metabolomic changes in the exocarp, mesocarp, and arils were measured using ^1^H NMR spectroscopy, and the results were analyzed using multivariate statistics. Results: Distinguishable differences were noted between the MAP and LPS conditions in fruit quality attributes and metabolite profiles. Sucrose levels in the aril, mesocarp, and exocarp samples were higher under LPS, while sucrose levels were reduced in MAP. In addition, alanine levels were more abundant in the mesocarp and exocarp samples, and ethanol concentration decreased in the exocarp samples, albeit less significantly. Conclusions: This pilot investigation shows the potential for using NMR as a valuable assessment tool for monitoring the performance of viable long-term storage conditions in horticultural commodities.

## 1. Introduction

Storing fruits for an extended period requires careful attention to their quality and preservation. Postharvest diseases and fungal pathogens pose significant risks to the quality of pomegranates (*Punica granatum* L.) when stored for extended periods. Although fungicides are widely used, their overuse may lead to pathogen resistance, rendering these agents ineffective over time [1]. Alternative preservation techniques, like modified atmosphere packaging (MAP) and controlled atmosphere (CA) cold storage, offer promising solutions for long-term storage [2].

Modified atmosphere packaging involves packaging in polymer films that maintain atmospheric pressure while reducing oxygen (O_2_) levels and increasing carbon dioxide (CO_2_) levels [3]. As the name suggests, the gas composition in a package is modified (not controlled) based on the respiration rate (O_2_ consumption, CO_2_ production) of the commodity inside to minimize microbial growth and chemical deterioration. This approach alters the gaseous composition within fruit packages to extend shelf life. Therefore, oxygen may be rapidly consumed when respiration rates increase due to storage temperature fluctuations, advanced senescence, or other factors, and the storage conditions can quickly become anaerobic. This poses a potential danger as anaerobic conditions can lead to fermentation, the degradation of fruit quality, and the development of off-flavors, allowing anaerobic pathogens to thrive.

Controlled atmosphere storage aims to maintain the optimum gas composition in a storage area within specified tolerances [4]. This method involves storage conditions that also feature reduced O_2_ and elevated CO_2_ concentrations in a controlled manner compared to regular air (RA). CA cold storage is more effective at preserving fruits and preventing decay than MA or RA storage. However, earlier postharvest pomegranate studies indicate that prolonged atmospheric cold storage can lead to browning, skin discoloration, and water loss from the juice into other fruit compartments [5]. Although CA storage can trigger anaerobic respiration due to diminished oxygen levels, maintaining an appropriate oxygen level can prevent this while reducing aerobic cellular respiration, which helps slow aging [1]. Both approaches can lower respiration and ethylene production rates, thereby delaying the physiological, pathological, and physical deterioration processes of the fruits.

Hypobaric or low-pressure storage (LPS) utilizes a controlled atmosphere for long-term preservation, achieved by using vacuum pumps to remove oxygen rather than adding nitrogen to displace it. This process rapidly reduces oxygen and pressure levels (within hours) to slow metabolism while maintaining sufficient oxygen to prevent anaerobic respiration, thereby extending the fruit’s postharvest shelf life [6,7,8,9,10]. Under LPS conditions, gas diffusion gradients are leveled, and the reduced number of air molecules allows for rapid gas exchange. Furthermore, it is hypothesized to prevent anaerobic conditions at even lower levels of O_2_ than what CA/MA technology can achieve [11]. These ultra-low O_2_ (ULO) conditions enable further reduction in respiration compared to CA/MA technologies.

At low-pressure conditions (below 101 kPa), a reduction of 10 kPa results in a 2% decrease in oxygen concentration, leading to lower rates of respiration and ethylene production [12]. Previous literature has stated that LPS efficacy initiates between 10 and 40 kPa, which can maintain O_2_ concentrations between 0.1 and 0.5% relative to regular air (RA) [13]. These ULO concentrations inhibit postharvest ripening (via ethylene biosynthesis reduction), fruit senescence, and extend fruit shelf life [14]. The LPS method has been applied to various fruits, including bananas, peaches, sweet cherries, loquat fruit, apples, and strawberries, to prevent reductions in fruit quality traits and physiological damage and enhance shelf life [7,14,15,16,17]. A recent detailed study by the authors presented a comprehensive evaluation of the technology in prolonging the postharvest life of blueberries [18,19].

Although several studies have demonstrated the benefits of LPS conditions for postharvest conservation, experimental details on the metabolomic changes under these conditions are limited. For instance, Xu and Li examined vibration injuries on pears under hypobaric conditions [20]. Therefore, in this pilot investigation, we present results from a metabolomics study using pomegranates as a model system.

Pomegranate is recognized as a superfood worldwide. Besides their economic benefits, pomegranates hold historical medicinal significance, as noted in many ancient texts [21,22]. There is growing interest in consuming pomegranate juice because of its nutritional benefits and perceived positive health effects [23,24,25,26]. Even the pomegranate exocarp (i.e., skin), often regarded as waste, has been shown to possess notable anticancer, antimicrobial, and antioxidant properties [27].

Traditional, non-invasive quality control methods involve physically inspecting fruit and assessing the levels of decay. A more objective and consistent approach consists of measuring the soluble solid concentration (SSC) or Brix, along with the percentage of titratable acid in the juice. These measurements are quick, semi-quantitative, and only sample the juice. Since storage conditions affect the entire fruit, methods capable of quantitatively analyzing all parts of the fruit are essential for comprehensive quality assessment and understanding postharvest biological processes. Additionally, we have recently shown that nuclear magnetic resonance (NMR)-based metabolomics can complement traditional methods. Here, we apply this technology to investigate the effects of LPS [28].

An NMR-based metabolomics study provides a robust metabolic framework for understanding plant phenotypic changes, facilitating the analysis of cellular processes in stored fruits [29]. Previous NMR studies have identified five sugars, five organic acids, and seven amino acids in pomegranates [30]. NMR has also been employed to evaluate other quality parameters affected by CA storage [31], including assessing the quality of pomegranates from various regions in Iran, the largest producer of pomegranates [32]. Additional methods, such as spectrophotometry and chromatography, have been used to detect chilling injury in pomegranates stored under cold conditions [33]. Although its sensitivity may be lower than these techniques, NMR provides simplified sample preparation and rapid, reliable quantification.

## 2. Materials and Methods

### 2.1. Experimental Design and Fruit Quality Analysis

Pomegranates were harvested and sourced from a commercial producer in California on 11 November 2021, and subjected to two postharvest treatments: low-pressure storage (LPS, <15 kPa) and modified atmosphere packaging (MAP). Each storage treatment had four replicates (four LPS chambers (RipeLocker, Inc., Bainbridge Island, WA, USA) and four macro-bins with MAP covers (StePac PPC, Buffalo Grove, IL, USA)) and were stored for five months at 7.2 °C. Storage environments were opened on 13 April 2022, and whole fruits were evaluated for weight loss and internal quality attributes (i.e., juice), as well as sampled for metabolomic analyses. Ten whole fruits were selected from each treatment replicate, and arils were extracted and homogenized into juice. Juice samples were then analyzed for pH, soluble solids concentration (SSC, %), titratable acidity (TA, as citric acid, %), and ethanol content (%) [18,34]. Quality analysis was conducted at harvest (Initial), immediately post-storage (i.e., Opening), and after 15 days of shelf life under ambient conditions (Shelf Life).

### 2.2. Metabolomics Sample Preparation

Pomegranate tissue samples (arils, mesocarp, exocarp) from each postharvest treatment (LPS and MAP) were divided into five replicates. Upon arrival, the samples were immediately prepared for processing. The pomegranate’s exocarp (outer skin) was peeled from the fruit, and the mesocarp (the white membrane tissues) was separated from the aril samples (seeds). The hard tissues, namely the mesocarp and exocarp, were ground using liquid nitrogen in a mortar and pestle while the arils were squeezed. Five exocarp, mesocarp, and aril sample replicates were prepared for each storage condition. The ground mesocarp and exocarp tissues were prepared by placing fifty grams of each replicate into a 5 mL Eppendorf tube and adding 3 mL of methanol. The aril samples were prepared by transferring 4 mL of each into a 4 mL centrifugal filter. After sonicating the hard tissues for 15 min, the aril, mesocarp, and exocarp samples were centrifuged at 18,000× *g* for 20 min. From each centrifuge filter unit containing the aril samples, 2 mL of filtrate was collected in a 1.5 mL Eppendorf tube with a 1 mm hole drilled in the cap, and the samples were then frozen. The supernatant of the centrifuged mesocarp and exocarp samples was collected in 5 mL Eppendorf tubes with two 1 mm holes drilled in the cap. After freezing all the samples, they were lyophilized for 48 h, with the 1 mm holes aiding solvent evaporation.

The samples were suspended in a D_2_O buffer solution containing 90 mM KH_2_PO_4_, 0.2 mM imidazole (as a pH indicator), and 0.05 mM sodium trimethylsilyl [2,2,3,3-d4] propionate (TSP, for chemical shift and concentration reference), and the pH was adjusted to 6.8. Following lyophilization, 700 µL of the prepared solution was added to the lyophilized samples, which were then vortexed for 20 s and centrifuged at 18,000× *g* for 20 min. The final 600 µL of the supernatant was transferred to pre-labeled 5 mm NMR tubes for further analysis. The methodology for the metabolomics sample preparation follows the protocols optimized in our laboratory [28,35,36]. All sample preparations were conducted over two days, and the samples were subsequently stored at 4 °C. All chemicals, reagents, and supplies were purchased from Fisher Scientific (Waltham, MA, USA).

### 2.3. NMR Experiments

Quantitative ^1^H-NMR spectra were recorded at 600 MHz using a JEOL NMR spectrometer equipped with a liquid N_2_-cooled probe and *Z*-axis pulsed field gradients at 300 K. One-dimensional ^1^H experiments involving mild pre-saturation of the water resonance were conducted with a 70° pulse angle (Ernst angle) [37,38]. NMR spectra were collected over 1024 transients, with an acquisition time of 2.0 s and a relaxation delay of 3.0 s.

**Figure A1:**
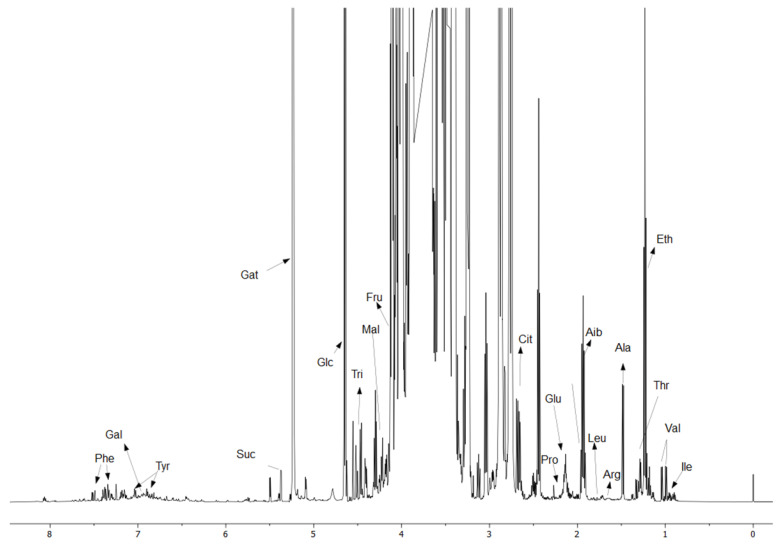
Representative ^1^H NMR spectra of the aril sample. The metabolites are identified as follows4-Aminobutyrate (Aib); Alanine (Ala); Arginine (Arg); Citrate (Cit); Ethanol (Eth); Fructose (Fru); Galactose (Gat); Gallate (Gal); Glucose (Glc); Glutamine (Glu); Isoleucine (Iso); Leucine (Leu); Malate (Mal); Phenylalanine (Phe); Proline (Pro); Sucrose (Suc); Threonine (Thr); Trigonelline (Tri); Tyrosine (Tyr); Valine (Val). Concentrations of the metabolites are listed in Table A1.

Data was preprocessed using MestReNova (version 14.3) by setting the spectrum size to 64 k, applying sine-squared 90° apodization, manually phasing, baseline correction, and normalizing and referencing the data to the TSP peak. The spectra were saved as .jdx files for metabolite identification using Chenomx. The spectra were processed and analyzed with Chenomx NMR Suite 8.1 software (2014, Chenomx Inc., Edmonton, AB, Canada). Fourier-transformed spectra were multiplied by an exponential weighting function corresponding to a line-broadening of 0.5 Hz. All spectra underwent manual phase correction and baseline optimization, with their chemical shifts referenced to the TSP standard. Although all experiments were conducted in D_2_O with mild pre-saturation of the residual H_2_O peak, the spectral region near the water resonance (±0.1 ppm) was excluded from metabolite peak identification. The resulting spectra were analyzed using the PROFILER Module of Chenomx, estimating the concentrations of selected metabolites across all samples. The combined concentration data were used for multivariate statistical analysis. The metabolite peaks from the processed spectra were analyzed and assigned to their chemical shifts using the built-in Chenomx tools. The assigned metabolites were compared and validated against chemical shift values from other ^1^H NMR-based metabolomics studies previously published [28,35,36]. The representative NMR spectrum is provided in the Appendix A (Figure A1).

### 2.4. Statistical Analysis of NMR Spectroscopy Datasets

**Figure A2:**
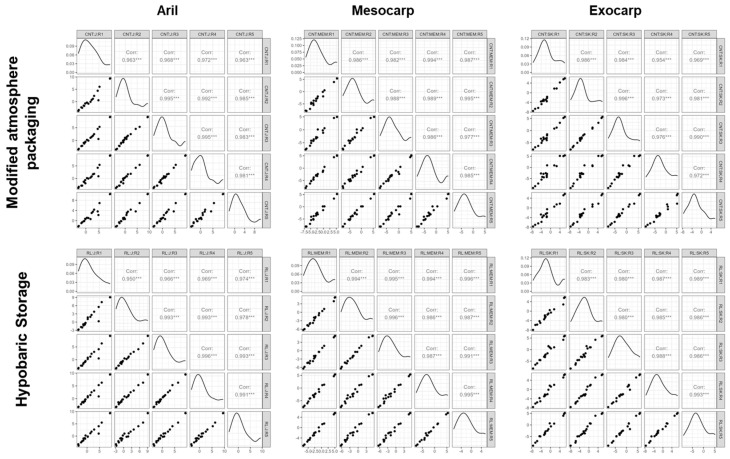
Correlation plot between the five replicates of each treatment (LPS (RL), MAP (CNT)) in each fruit tissue (Aril, Mesocarp, Exocarp).

Fruit quality attributes and weight loss were assessed for storage technology treatment comparisons at each time point using an ANOVA and means comparison tests, including Tukey HSD and pooled *t*-test, in JMP version 18 (*p*-value < 0.05). For metabolomic analysis, a well-established multivariate statistical analysis approach was utilized to identify differentially altered metabolites. Statistical methods rely on established protocols within R (version 4.0) statistical procedures and have been previously applied to metabolomics and other analyses [28,35,36,39]. The replicates for each experimental condition demonstrate a correlation coefficient of greater than 0.97 among the samples (Figure A2).

Metabolites that are differently changed between groups are identified using linear modeling with the LIMMA package. The control group includes the exocarp, mesocarp, and aril of fruits stored under MAP. The other groups contain the same tissues but are stored under LPS. Lists of metabolites showing the most significant differences between groups (MAP vs. LPS for each tissue: exocarp, mesocarp, and aril) are generated. Significantly altered metabolites were selected through a two-step process. First, the initial dataset included all metabolites with a detectable signal in at least one comparison (e.g., MAP vs. LPS for aril samples). Second, data from all comparisons were combined into a single dataset. This combined dataset included metabolites that showed changes in at least one experimental comparison. An F-test identified differential measurements within groups. *p*-values for each analyte were adjusted for multiple comparisons using the false discovery rate (FDR) adjustment (FC > 1.2 and *p*-value < 0.05) with the Benjamini–Hochberg procedure [40,41]. Fold changes were calculated through multivariate statistical analysis. The significance threshold was a *p*-value less than 0.05 and a fold change (log2) greater than 1.5, unless specified otherwise in specific analyses. All analyses and plots were generated using a combination of Bioconductor (version 3.1) and R (version 4.0) [42,43].

## 3. Results and Discussion

### 3.1. Fruit Quality and Metabolites Broadly Differentiate Between Storage Conditions

Anthony et al. recently evaluated the commercial efficacy of LPS in extending the storage period of blueberries [18,34]. They demonstrated benefits in reducing weight loss and softening, suppressing fungal mold and decay, and maintaining internal fruit quality [16]. After extended storage conditions, weight loss was significantly different between the two storage treatments (Table 1). Under LPS conditions, where the air is fully saturated, weight loss was significantly reduced. In contrast, the fruit stored in MAP demonstrated significantly higher weight loss values, possibly due to lower relative humidity levels inside the bag compared to the LP environment (Table 1). This reduced weight loss under LP conditions has been demonstrated in previous research on blueberries [18]. When comparing the internal quality attributes of the pomegranate juice, no differences were observed in SSC, except at the initial and subsequent time points, regardless of the storage treatment. At the same time, TA was higher in the LPS treatment at both time points (Opening and Shelf Life). Although not significantly different, this demonstrated reduced substrate utilization, possibly due to the inhibitory effects of the LP environment on respiration and metabolic activities (Table 1). As a result, the LPS fruit maintained a lower SSC/TA ratio, which was more similar to the at-harvest (Initial) levels than the MAP control (Table 1). However, given the extremely low oxygen environment of the LP chambers, this may have contributed to increased periods of anoxia and fermentation induction, contributing to slightly elevated levels of ethanol in LPS versus MAP fruit (Table 1).

**Figure 1:**
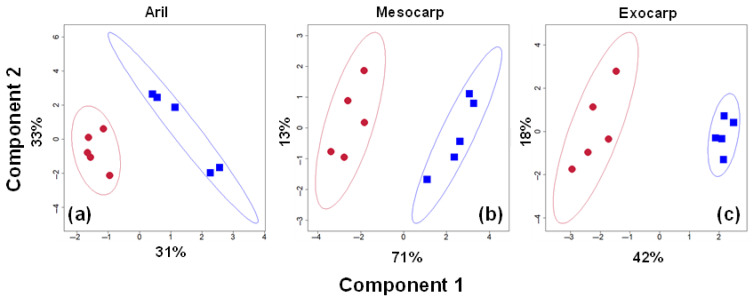
PLS-DA (partial least squares discriminant analysis) metabolomic differentiation between the modified atmospheric package (blue) and the low-pressure storage conditions (red). Classification of (**a**) aril, (**b**) mesocarp, and (**c**) exocarp samples. Each dot represents one of the replicate measurements, and ellipses denote the 95% confidence region over the classifications. Components 1 and 2 are plotted along the *X* and *Y* axes, respectively, with their respective variances.

A total of twenty metabolites were identified in all the samples (Table A1). Specifically, four sugars, ten amino acids, four organic acids, and two other metabolites were identified. Nineteen of the identified metabolites were detected in the aril samples, while only eighteen metabolites were detected in the hard tissues, specifically the mesocarp and exocarp. Figure 1 shows the partial least squares discriminant analysis (PLS-DA) for the aril (Figure 1a), mesocarp (Figure 1b), and exocarp (Figure 1c) samples. The five replicate samples under modified atmospheric package (MAP) conditions (blue symbols) and low-pressure storage (LPS) conditions (red symbols) were distinguished from all the other samples. Table A1 contains metabolite concentration (mM) data collected from the aril, mesocarp, and exocarp samples. A correlation analysis of the five replicates yields a correlation coefficient greater than 0.96 (representative analysis of the aril, mesocarp, and exocarp samples, as shown in Appendix A Figure A2).

### 3.2. Differential Alterations of Metabolites Due to Low-Pressure Storage

As a next step, particular metabolites that are differentially altered due to LPS compared to the MAP (as a control) are evaluated using a multivariate approach. For each sample condition (aril, mesocarp, or exocarp), a metabolite is altered significantly if the |fold-change| (FC) is more significant than 1.5 and the FDR-adjusted *p*-value is <0.05. The relative fold changes are given in Table 2, and the concentrations (average ± standard deviation) are listed in Table A1.

**Figure A3:**
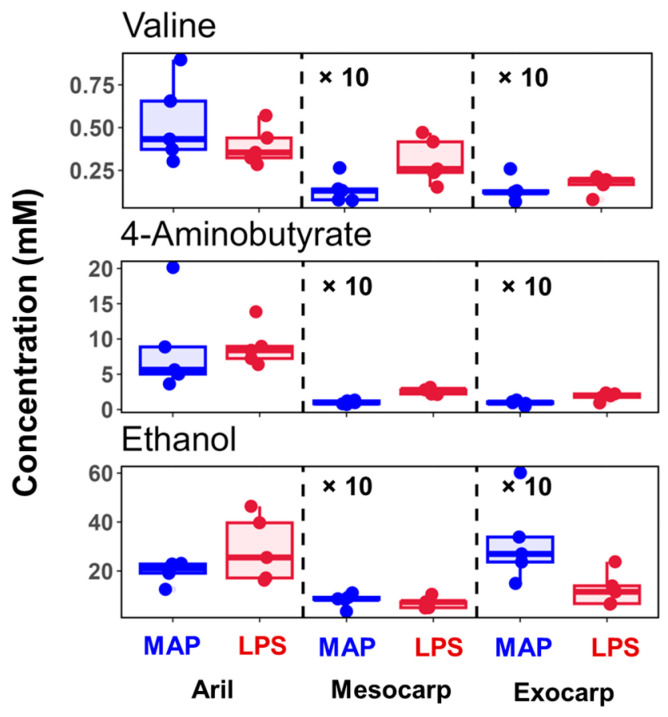
Metabolites in low-pressure storage (LPS) compared to modified atmospheric package (MAP) conditions. The box–whisker plots of valine, 4-Aminobutyrate, and ethanol in aril, mesocarp, and exocarp samples. The mesocarp and exocarp concentrations are scaled (×10).

Under LPS conditions, the sucrose levels were significantly higher in aril, mesocarp, and exocarp samples (Figure 1a). The aril samples show a downregulation of the amino acids proline, glutamine, and arginine, as well as the organic acid malate (with *p*-value < 0.05). In the mesocarp samples, alanine exhibits a significant increase in LPS conditions (fold change > 2 and *p*-value < 0.001) (Figure 1b). In contrast, 4-aminobutyrate (GABA), valine, isoleucine, and leucine levels increase to a lesser extent (*p*-value < 0.05). In the case of exocarp samples, ethanol levels are notably lower (fold change ~1.4 times) with a less significant increase in 4-aminobutyric acid (GABA). While in the arils, ethanol is slightly higher (albeit non-significant) in the LPS treatment, which supports the quality analysis results as well (Table 1). Figure A3 compares the changes in valine, 4-aminobutyrate (GABA), and ethanol between the conditions.

A potential list of metabolites detected in pomegranate juice samples has been published [44,45,46]. Additionally, Tang and Hatzakis presented a comprehensive NMR-based metabolomics study of pomegranate juices from three different cultivars, in which 19 metabolites were quantified using ^1^H and ^13^C NMR spectroscopy [30]. Between the two studies, ten metabolites were detected in common, while five metabolites were not detected in this study (arabinose, aspartic acid, formic acid, punicalagin, and succinic acid). One likely reason is that the previous work utilized two-dimensional NMR experiments at a higher spectrometer frequency.

### 3.3. Representative Changes in the Metabolites Under LPS Conditions

**Figure 2:**
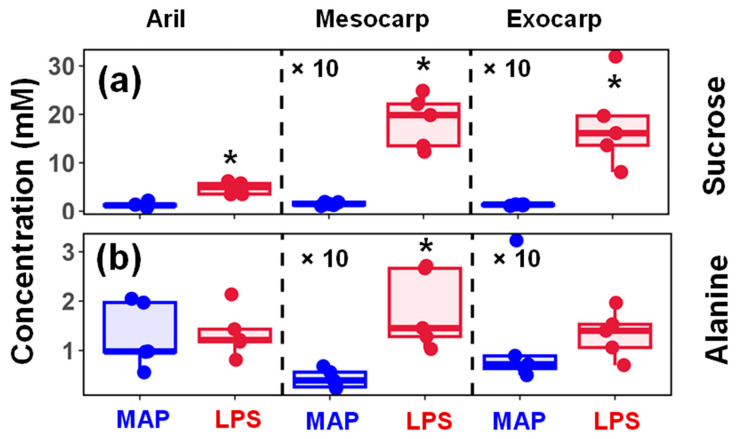
Metabolites of high significance in low-pressure storage (LPS) compared to modified atmospheric package (MAP) conditions: box–whisker plots of (**a**) sucrose and (**b**) alanine concentration in aril, mesocarp, and exocarp samples. The mesocarp and exocarp concentrations are scaled (×10). Metabolites that show significant changes (|fold-change| > 1.5 and *p*-value < 0.05) with respect to the MAS are marked with *.

Postharvest storage conditions can induce extensive and diverse changes in the transcriptome and metabolome, leading to a significant reorganization of metabolism and cellular function. In particular, the variations in the metabolome can be used to understand the metabolic pathways altered under specific postharvest conditions [47]. In the current study, sucrose levels tended to be higher (two to four times) in all samples under LPS conditions (Figure 2). It has been previously noted that sucrose content tends to decrease during postharvest ripening [48]. Additionally, in a comprehensive study, Fugate et al. noted endogenous metabolism-mediated losses in sucrose content and processing quality in postharvest sugar beet roots [49]. This data supports the hypothesis that postharvest metabolic processes, such as sucrose hydrolysis and other enzymatic degradation, are inhibited during LP storage.

## 4. Discussion

Our results indicate significantly higher sucrose concentrations in aril (~two-fold), mesocarp (~3.6 times), and exocarp (~3.6 times) samples (Table 2 and Figure 1), with no significant difference (*p*-value > 0.05) in glucose or fructose levels (Table A1). The dynamics of carbohydrate metabolism in cold-stored fruits have been extensively investigated, revealing differential accumulation patterns of individual sugars, with a general trend toward elevated sucrose concentrations [47,50]. In contrast, our prior research on the temporal modulation of citrus fruit under cold storage demonstrated a decline in sucrose levels after the fourth week [28].

Under LPS conditions, three other metabolites show changes in one of the samples (aril, mesocarp, or exocarp). These are alanine, 4-aminobutyrate (GABA), and ethanol (Table 2 and Table A1, Figure 1 and Figure A1). The LP storage of pomegranates indicates a significant increase in alanine (>2-fold, *p*-value < 0.05) only in the mesocarp (Table 2 and Figure 1). In addition, the mesocarp tissues show slightly elevated concentrations of 4-aminobutyrate (GABA) and decreased levels of ethanol only in the exocarp samples (Table A1). Alanine is one of the primary metabolites related to hypoxia in many plants and fruits [47,48,51,52,53,54,55]. The accumulation of alanine and GABA appears to be a typical response of plants to hypoxia and is highly reactive and sensitive to changes in oxygen and carbon dioxide levels [56]. GABA contents have also increased in strawberries and tomatoes during postharvest carbon dioxide treatment [57,58]. Furthermore, as the accumulation of GABA depends on oxygen concentrations, it is considered a hypoxia marker in pears [52,59]. Studies on apples suggest that alanine concentrations increase after low-oxygen-induced stress, which differs from the levels of ethanol [52]. Low-pressure storage modulates respiratory activity via ultra-low oxygen conditions, which is a critical determinant of postharvest metabolism, and elicits both a reduction in sucrose hydrolysis and a potential spike in anaerobic byproducts (alanine, GABA, ethanol). Therefore, the LP conditions evaluated in this study may have had O_2_ levels set a bit too low, which resulted in a period of anoxia throughout storage, as reflected in the metabolite profiles.

A fundamental physiological response to hypoxia in fruits is the shift from aerobic to anaerobic respiration, serving as a compensatory mechanism for energy depletion. This low-oxygen environment directly impacts the activity and gene expression of enzymes involved in sugar metabolism. However, monosaccharides’ quantitative changes and metabolic fate (glucose, fructose) and sucrose under LPS remain incompletely characterized. A complex interplay of environmental factors, including oxygen and carbon dioxide partial pressures, temperature, storage duration, and inherent genotypic variation, can influence these metabolic responses. Other factors that can cause variability in metabolic profiles include the harvest year, growing conditions, rootstocks, and underlying abiotic and biotic stresses [60,61,62]. Further experiments involving genomic or proteomic studies are necessary to understand the biochemical pathways responsible for the observed changes in metabolite levels.

Overall, the lower sucrose levels in modified atmosphere packaging (MAP) may have been a result of higher respiration rates (when compared to LPS), as sucrose is typically broken down into monosaccharides throughout storage. Plants start with elevated sucrose levels, absorbing these larger, complex sugars in the fruit tissue during growth and development. However, these complex carbohydrates break down to support metabolic activities during storage. It is hypothesized that this breakdown occurred more rapidly in MAP compared to LPS due to the increased respiration rates in the control group versus the LPS treatment.

## 5. Conclusions

NMR-based metabolomics could serve as a tool to monitor biomarkers under variable postharvest storage regimes. In particular, sucrose emerged as a sensitive metabolite that can be used as an indicator of pomegranate storage quality. NMR spectroscopy has not been widely used for postharvest examinations of fruits. However, this study demonstrates the potential for NMR techniques to be considered a complementary tool for real-time respiration monitoring, potentially aiding in postharvest storage decisions. Several metabolic trends were observed in different tissues, specifically the arils, mesocarp, and exocarp. The results demonstrate that metabolites such as sucrose, alanine, or ethanol are sensitive to LPS conditions, broadly indicating the feasibility and potential of metabolite profiling as an alternative approach to monitoring postharvest storage. However, the same trends will be monitored for other fruit species and varieties under identical experimental conditions, and additional experiments are warranted. Even fruits of the same species or variety harvested from different geographic regions can be expected to exhibit distinct metabolite profiles. Nonetheless, the results herein have demonstrated that NMR spectroscopy can identify specific metabolic trends of pomegranate under various storage conditions.

## Figures and Tables

**Table 1:** Internal fruit quality attributes of pomegranate juice and whole fruit weight loss (%) at harvest (initial) and after storage and shelf life periods, from two postharvest storage technologies (low-pressure storage (LPS) and modified atmosphere packaging (MAP)).

Time Point	Storage Treatment	pH	SSC ^a^ (%)	TA ^b^ (%)	SSC/TA Ratio	Ethanol (%)	Weight Loss (%)
0 d (Initial)	Initial	3.35 ± 0.04 ^b^	17.1 ± 0.47 ^a^	1.09 ± 0.01 ^a^	15.7 ± 0.58 ^a^	0.01 ± 0.00 ^b^	-
212 d (Opening)	LPS	3.67 ± 0.07 ^a^	15.9 ± 0.34 ^b^	0.84 ± 0.06 ^b^	18.9 ± 1.24 ^abc^	0.44 ± 0.06 ^a^	0.6 ± 1.4 ^b^
	MAP	3.72 ± 0.04 ^a^	15.6 ± 0.54 ^b^	0.72 ± 0.05 ^b^	21.8 ± 1.42 ^a^	0.23 ± 0.13 ^ab^	3.6 ± 1.8 ^a^
+15 d (Shelf Life)	LPS	3.63 ± 0.09 ^a^	15.6 ± 0.64 ^b^	0.88 ± 0.10 ^b^	17.9 ± 2.21 ^bc^	0.50 ± 0.23 ^a^	-
	MAP	3.60 ± 0.09 ^a^	15.2 ± 0.63 ^b^	0.74 ± 0.08 ^b^	20.8 ± 2.54 ^ab^	0.31 ± 0.23 ^ab^	-
Significance		*	*	*	*	*	*

Means ± SD are displayed. ^a^ Soluble solids concentration; ^b^ titratable acidity (citric acid). Significance according to ANOVA (*p*-value = * < 0.05, ns = nonsignificant). Means with different letters indicate a significant difference at *p* < 0.05 according to Tukey HSD or pooled *t*-test (for weight loss).

**Table 2:** Metabolites were significantly altered due to storage conditions. Positive fold change (FC) indicates up-accumulation in low-pressure storage (LPS), while a negative FC indicates down-accumulation.

Metabolites	Aril	Mesocarp	Exocarp
	^a^ FC	^a^ FC	^a^ FC
Sucrose	**1.9**	**3.58**	**3.65**
Proline	**−1.04**	−0.02	−0.22
Malate	**−0.79**	0.93	0.08
Glutamine	**−0.91**	0.21	0.39
Arginine	**−0.59**	0.43	0.34
Alanine	0.15	**2.12**	0.45
4-Aminobutyrate	0.28	**1.39**	**0.96**
Valine	−0.36	**1.2**	0.33
Isoleucine	−0.35	**1.12**	0.59
Leucine	−0.05	**0.89**	−0.08
Ethanol	0.46	−0.18	**−1.38**

^a^ FC: Fold-change between low-pressure storage (LPS) and modified atmospheric package (MAP); Underlined bold numbers indicate a *p*-value < 0.05.

## Data Availability

All data generated or analyzed during this study are included in this published article and files found in the Appendix A.

## Appendix A

**Table A1:** Metabolite concentrations (mM, average ± standard deviation) in both storage treatments (LPS, MAP) from each fruit tissue (aril, mesocarp, exocarp).

Metabolites	Aril		Mesocarp		Exocarp	
	MAP (mM)	LPS (mM)	MAP (mM)	LPS (mM)	MAP (mM)	LPS (mM)
4-Aminobutyrate	8.66 ± 5.99	8.96 ± 2.61	0.1 ± 0.02	0.26 ± 0.04	0.09 ± 0.03	0.18 ± 0.05
Alanine	1.31 ± 0.6	1.35 ± 0.44	0.04 ± 0.02	0.18 ± 0.07	0.12 ± 0.1	0.13 ± 0.04
Arginine	0.88 ± 0.41	0.54 ± 0.02	0.07 ± 0	0.1 ± 0.03	0.16 ± 0.05	0.2 ± 0.03
Citrate	60.83 ± 31.23	84.78 ± 39.11	17.43 ± 4.73	25.26 ± 6.09	16.51 ± 10.84	24.51 ± 13.8
Ethanol	19.7 ± 3.88	29.01 ± 12.12	0.81 ± 0.25	0.7 ± 0.2	3.19 ± 1.54	1.25 ± 0.63
Fructose	783.51 ± 339.42	626.91 ± 52.52	37.16 ± 3.68	41.05 ± 4.83	55.22 ± 8.79	63.46 ± 18.21
Galactose	7.44 ± 10.21	6.42 ± 9.59	0.38 ± 0.36	0.21 ± 0.03	0.72 ± 0.61	1.01 ± 0.92
Gallate	-	-	0.04 ± 0.01	0.05 ± 0.01	0.11 ± 0.04	0.17 ± 0.07
Glucose	728.77 ± 312.72	639.8 ± 63.43	35.95 ± 3.67	38.99 ± 5.76	39.9 ± 5.26	51.72 ± 15.18
Glutamine	3.75 ± 2.4	1.82 ± 0.82	0.5 ± 0.22	0.56 ± 0.18	0.24 ± 0.07	0.31 ± 0.09
Isoleucine	0.14 ± 0.05	0.11 ± 0.03	0.01 ± 0	0.01 ± 0	0.01 ± 0	0.01 ± 0
Leucine	0.12 ± 0.05	0.1 ± 0.02	0.01 ± 0	0.01 ± 0	0 ± 0	0 ± 0
Malate	5.43 ± 3.41	2.68 ± 0.55	1.36 ± 0.14	2.62 ± 0.46	2.27 ± 0.73	2.44 ± 0.94
Phenylalanine	0.54 ± 0.18	0.41 ± 0.06	-	-	-	-
Proline	1.86 ± 0.88	0.84 ± 0.18	0.14 ± 0	0.15 ± 0.04	0.14 ± 0.09	0.11 ± 0.03
Sucrose	1.32 ± 0.47	4.79 ± 1.1	0.15 ± 0.03	1.85 ± 0.49	0.13 ± 0.01	1.79 ± 0.8
Threonine	0.32 ± 0.18	0.21 ± 0.04	0.03 ± 0	0.04 ± 0.01	0.07 ± 0.02	0.05 ± 0.01
Trigonelline	0.8 ± 0.41	0.62 ± 0.11	0.05 ± 0.01	0.06 ± 0.01	0.04 ± 0.02	0.04 ± 0.01
Tyrosine	0.28 ± 0.13	0.26 ± 0.06	-	-	-	-
Valine	0.53 ± 0.22	0.39 ± 0.1	0.01 ± 0.01	0.03 ± 0.01	0.01 ± 0.01	0.02 ± 0

MAP (Modified atmospheric package); LPS (low-pressure storage).
